# Establishing a New ECMO Referral Center Using an ICU-Based Approach: A Feasibility and Safety Study

**DOI:** 10.3390/healthcare10030414

**Published:** 2022-02-22

**Authors:** Ryszard Gawda, Maciej Piwoda, Maciej Marszalski, Katarzyna Lyp, Jolanta Piwoda, Magdalena Maj, Maciej Gawor, Maciej Molsa, Marek Pietka, Tomasz Czarnik

**Affiliations:** 1 Department of Anesthesiology, Intensive Care and Regional ECMO Center, Institute of Medical Sciences, University of Opole, 45-401 Opole, Poland; mpiwoda@mp.pl (M.P.); maciek.marszalski@gmail.com (M.M.); molsamaciej@gmail.com (M.M.); tczarnik@mac.com (T.C.); 2 Department of Family Medicine and Public Health, Faculty of Medicine, University of Opole, 45-052 Opole, Poland; katarzyna.lyp@uni.opole.pl; 3 Department of Anesthesiology, Intensive Care and Regional ECMO Center, University Hospital in Opole, 45-401 Opole, Poland; jo_linka@interia.pl (J.P.); truchan.m@gmail.com (M.M.); macgawor@interia.pl (M.G.); mpietka@gmail.com (M.P.)

**Keywords:** critical care, healthcare, intensive care unit, extracorporeal membrane oxygenation, ECMO

## Abstract

Background: A high-volume center with a multidisciplinary team is regarded as the optimal place for providing extracorporeal membrane oxygenation (ECMO). We hypothesize that an ECMO center can also be successfully created and subsequently developed entirely by intensivists in a mid-size mixed intensive care unit (ICU). Methods: A model was created for setting up a new ECMO referral center within the structure of an existing mixed ICU in a tertiary hospital. A retrospective analysis was carried out of the first 33 patients treated in the initial period of the center’s activity, from mid 2018 to the end of 2020. Results: An ECMO center was established and developed entirely based on the resources of an existing mixed ICU. Thirty-three patients were treated. They had an overall survival rate at 90 days of 60.6%. In veno-venous (VV) mode ECMO duration, ICU length of stay, and SOFA score were significantly higher than in veno-arterial mode. No significant differences in clinical characteristics were observed between survivors and non-survivors on VV-ECMO. Conclusions: A regional ECMO center can be set up as an integral part of a mixed ICU in a tertiary hospital. Extracorporeal therapy, such as continuous renal replacement therapy and mechanical ventilation can be managed entirely by intensivists. Further studies are needed to show that the ICU-based approach to setting up a new ECMO center is no less effective than the multidisciplinary approach.

## 1. Introduction

Extracorporeal membrane oxygenation (ECMO) is one of the most advanced techniques used in acute respiratory, as well as circulatory, failure [1]. In 2020, 492 ECMO centers were registered at the Extracorporeal Life Support Organization (ELSO), the largest worldwide organization consociating health care workers involved in extracorporeal therapy [2]. Experts argue that the optimal conditions for this kind of treatment are best provided in high-volume dedicated centers, since the outcomes are linked with the volume of patients [3,4]. In reality, the majority of ECMO procedures are performed in low-volume centers [5,6].

There are numerous requirements to fulfil to successfully set up a new ECMO center. Obtaining an institutional commitment and financial support seems to be one of the most significant. How to form an ECMO team and where to locate a new ECMO center in a hospital area is another fundamental problem. Additionally, recommendations suggest creating a multidisciplinary group consisting of various specialists, because of the complicated nature of extracorporeal therapy [7,8,9,10].

Reports on practical methods for setting up new ECMO centers are limited [11,12]. To date, there have been no reports describing the establishment of ECMO centers solely from the perspective of an individual mixed intensive care unit (ICU). We hypothesize that an ECMO center guided only by intensivists has the potential to be a full value ECMO referral center. Our primary aim was, thus, to describe the main steps required for the establishment of a new ECMO center and to present a concept of an ICU-based approach to conducting ECMO therapy. Our secondary aim was to outline the patient demographic data and outcomes achieved in the initial period of the center’s activity.

## 2. Materials and Methods

This is a feasibility and safety study on the setting up of a new ECMO referral center in a tertiary hospital. The study design was approved by the Regional Ethics Committee (date of approval: 11 June 2021, decision number: 335/2021). The requirement for patient written informed consent was waived.

Our ECMO center was established solely as a part of the existing mixed 11-bed ICU of the University Hospital in Opole, Poland. This is a level 3 ICU with intensivists constantly present, the nurse to patient ratio is 1:2, and the full spectrum of organ dysfunction is treated. Besides the mixed ICU, critically ill patients in our hospital are also treated in the cardiosurgical ICU and pediatric/neonatal ICU. These ICUs are administratively separate structures. Staff at our ICU consist only of intensivists, registered nurses, and physiotherapists. The ICU serves approximately 450 patients a year.

When our ECMO center was set up, there was no coordinated ECMO system in Poland. Moreover, ECMO therapy was practically inaccessible in Opole Voivodeship. No specific financial support for the ECMO program was initially allocated, except for funds to purchase the first ECMO machine. The institutional commitment was mainly limited to the approval of administrative routines. All ECMO procedures are financed intrinsically by the Polish National Health Fund.

**Figure 1:**
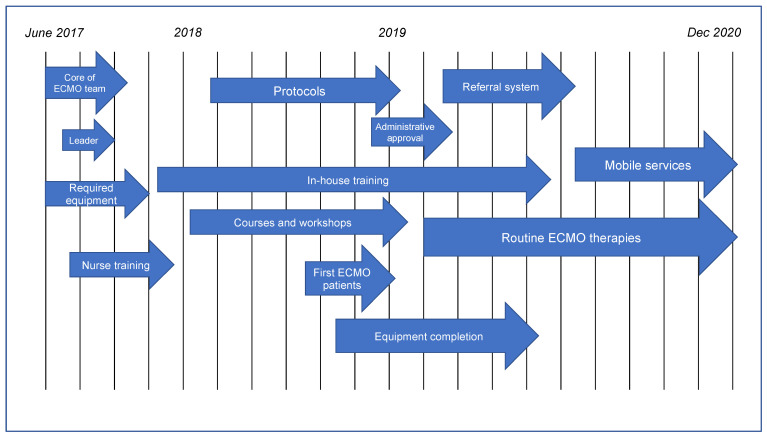
Timeline of the ECMO center progression.

Chronologically, the overall development of the ECMO center was divided into four main steps: initialization, training, preparation, and activation. A timeline of the center’s progress is shown in Figure 1.

### 2.1. Initialization

The concept of establishing an ECMO program was raised at the beginning of 2017 by two intensivists (T.C. and R.G.) before scheduled updating of the ICU equipment. After institutional approval, a sum of EUR 90,000 was allocated for the purchase of an ECMO machine (Cardiohelp, Maquet, Germany) and to finance intensivists attending simulation workshops in November 2017 (Rastatt, Germany). A group of six board-certified intensivists of the existing ICU formed the ECMO team, and a leader (T.C.) was designated.

### 2.2. Training

In the first half of 2018, the ECMO team attended numerous internal training sessions, including (a) thorough theoretical preparation, (b) ECMO machine management, including basic servicing and priming a circuit, and (c) simulation of typical critical scenarios. The registered nurses of our ICU attended ECMO courses held exclusively for ECMO nurses and were subsequently trained by the ECMO team physicians. During 2018, team members attended courses and workshops in external centers, including the ELSO ECMO course at Karolinska University Hospital (Stockholm, Sweden), Paris Rescue Team Course (Paris, France), and ECMO Simulation Workshops (Poznan, Poland).

### 2.3. Preparation

In the second half of 2018, the ECMO team announced that they were ready to perform ECMO therapy on some selected patients hospitalized in the University Hospital. Two veno-venous ECMO (VV-ECMO) and two veno-arterial ECMO (VA-ECMO) therapies were conducted to the end of 2018. Internal training was continued in 2018 and a second ECMO machine (Cardiohelp, Maquet, Germany) was bought with financial support from the local government.

### 2.4. Activation

Technically, the local government and hospital authorities inaugurated the Regional ECMO Center in Opole in January 2019. A short local media campaign was conducted, and the district hospitals were informed of the center’s activation. The center served a population of the Opole area (one million citizens). At the end of 2019, the center extended its activity to patients from adjacent regions. Mobile services, including both ground transportation (at the beginning of 2020) and a helicopter (at the end of 2020), were thus introduced. Finally, a third ECMO machine was bought.

**Figure 2:**
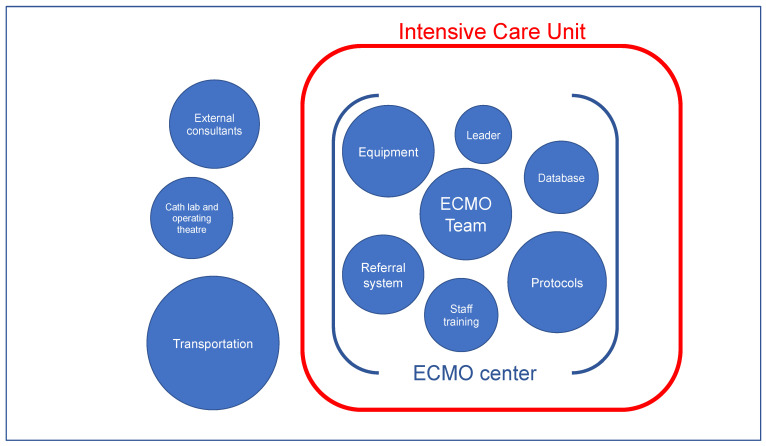
Components of the ICU-based ECMO center.

Establishing and developing an ECMO center was complex. The most significant milestones of the ECMO center activity are shown in Figure 2.

### 2.5. Protocols

To qualify for VV-ECMO, we incorporated criteria used in the EOLIA trial as core indications [13]. However, the final patient qualification is individualized and based on the clinical context (actual clinical data of the patient or on existing therapy trends). Several internal protocols were incorporated into daily practice, including plans of mechanical ventilation, patient’s weaning, anticoagulation proceedings, and pharmacotherapy adjustment. Protocols for critical situations (oxygenator dysfunction, extracorporeal system failure, accidental decannulation, recirculation) during ECMO were prepared and practiced. Protocols for intra- and inter-hospital ECMO transportations were also prepared.

### 2.6. Patient Treatment

VA-ECMO is accessible only for in-hospital patients. Cannulation is performed percutaneously both by ECMO intensivists in the ICU and collectively with interventional cardiologists in the catheterization laboratory using real-time ultrasound (US) guidance. In the case of indications of left ventricle unloading, an Impella device or intra-aortic balloon pump are inserted percutaneously by an interventional cardiologist. An arterial cannula is only removed in the operating theatre by a vascular surgeon.

VV-EMCO is provided both for in- and out-of-hospital patients. Before cannulation, an ECMO intensivist performs a routine transthoracic echocardiogram (TTE) and ultrasound examination of both the lungs and great vessels. Cannulation is performed solely percutaneously using real-time US-guidance, and the position of the pericardiac cannula is confirmed by an ECMO intensivist with the use of TTE or a transesophageal echocardiogram (TEE).

Both methods of extracorporeal therapy are conducted in the ICU. Only intensivists (ECMO team members) are responsible for all aspects of the extracorporeal therapy, e.g., priming the circuit, patient monitoring and examination, ECMO machine handling, treatment assessment, weaning, and procedure termination. Anticoagulation is provided with continuous administration of unfractionated heparin. Protective mechanical ventilation is applied to maintain a tidal volume below 6 mL/kg of predicted body weight with plateau pressure below 25 cm H_2_O, and positive end-expiratory pressure around 10 cm H_2_O. All routine procedures of up-to-date intensive care are provided, including daily rounds, patient-tailored feeding, sedation holiday, and patient early mobilization. After decannulation, the patients are treated accordingly, still in the same ICU, by ECMO team members, until the patients can be safely transferred out of the intensive care environment.

### 2.7. Staff and Equipment

All extracorporeal therapies are managed solely by intensivists who are members of the ECMO team. While, 10 intensivists have been trained and are fully involved in the ECMO center activity. The ECMO team is supported by registered nurses working at the bedside in the ICU. As in other critically ill patients, physiotherapists are involved in the patient’s early mobilization. No additional health professionals (perfusionists, surgeons, cardiologists, respiratory therapists, nutritionists, pharmacists, etc.) are involved in the extracorporeal therapy. A vascular surgeon, interventional cardiologist, and cardiac surgeon can be called upon at an hour’s notice if required.

### 2.8. Referral System

A referral system for patients treated outside the University Hospital in Opole was created. This is based both on a website and two dedicated telephone lines. The data on the reported patients are analyzed collectively by at least two ECMO team physicians and the final decision is taken within 30 min. Four resolutions are then applied: (1) qualification with ground or rotor-wing transportation of the patient executed by an emergency medical service (EMS), (2) qualification and activation of the mobile ECMO team, activating the ECMO procedure at the referring hospital with subsequent transportation on ECMO either by ground or air, (3) temporal disqualification with recommendations regarding optimization of therapy and repeated consultation the next day, (4) ultimate disqualification. The COVID-19 pandemic imposed a specific approach for the qualification of SARS-CoV-2 infected patients, in line with specific ELSO guidelines [14].

### 2.9. ECMO Transportation

For transportation, strict cooperation with the EMS was established. The mobile ECMO team consisted of two ECMO team intensivists (alternatively an ECMO intensivist with residency in intensive care medicine) and two paramedics from the EMS. An ambulance or helicopter is provided by EMS. To date, the mobile ECMO team has serviced patients only on VV-ECMO. Currently, there is no coordinated cardiogenic shock referral system in Opole Voivodeship. The ECMO team is equipped with everything necessary for ECMO commencement including an ECMO machine, cannulas, respirator, and an ultrasound machine with TEE probe. After arrival at the referring ICU, the ECMO team performs cannulation and transfers the patient started on extracorporeal therapy to the Regional ECMO Center in Opole. Members of the ECMO team are insured against unforeseen accidents. During the COVID-19 pandemic, our mobile ECMO team performed ground and rotor-wing aircraft transportation of SARS-CoV-2 infected patients on VV-ECMO.

### 2.10. Database

All patients are entered anonymously into two separate databases created as Excel spreadsheets. The first database comprises patients started on VA-ECMO therapy. The second database comprises patients treated with VV-ECMO. Patient baseline data are collected on therapy, complications, and outcomes. A phone-based follow-up of every patient is conducted to assess the outcomes at 28 and 90 days after decannulation. Every patient is also recorded on the ELSO registry.

### 2.11. Statistical Methods

Data analysis was performed using Statistica 13.3 PL (TIBCO Software Inc., Palo Alto, CA, USA). For continuous variables, the median and IQR *Q*_1_–*Q*_3_ were calculated, while the discrete variables were expressed as percentages, and cardinality (*n*) was taken into account and verified with the $x^{2}$ test. The normality of the distribution was verified using the Shapiro–Wilk test. In the case of non-compliance of the distribution of variables with the normal distribution, the Mann–Whitney U test (with corrections for continuity) was used, and in the remaining cases, the T test was applied. Statistical differences were considered significant at *p* < 0.05.

## 3. Results

The development of all stages of the Regional ECMO Center in Opole have been completed within 28 months. As a result, VV-ECMO therapy is now provided for the population of approximately 3 million. VA-ECMO therapy is ensured only for the patients of the University Hospital. Both methods of extracorporeal therapy are performed entirely within the existing mixed ICU and are conducted only by intensivists. This way, ECMO is considered as another modality in the ICU, alongside mechanical ventilation and renal replacement therapy. Availability of on-call cardiovascular specialists and participation of EMS in ECMO transportation are the only constituents of our ECMO center that are derived from the outside of the ICU.

From July 2018 to December 2020, 33 patients were treated: 22 on VV-ECMO (including eight cases of COVID-19) and 11 on VA-ECMO. Baseline characteristics of all patients are presented in Table 1. Median support time on VV-ECMO was 365 h, which was significantly higher than the median time of 49 h on VA-ECMO. Significant differences were also observed between the VV-ECMO and VA-ECMO groups regarding SOFA score and ICU length of stay. Overall survival rate at ICU discharge was 63.6%, survival rate at 28 and at 90 days after decannulation was 63.6% and 60.6%, respectively.

Characteristics of the VV-ECMO therapy are shown in Table 2. No significant differences were observed between survivors and non-survivors. A main indication for ECMO was acute respiratory distress syndrome (ARDS) caused by viral (COVID-19, 8 patients, influenza, 2 patients) or bacterial infection (8 patients). In four cases of ARDS, pneumonia was of unknown etiology. The complications during VV-ECMO were as follows: nasopharyngeal bleeding (4 patients), pneumothorax (2 patients), and bleeding from cannulation sites (one patient). Four patients were cannulated in the external hospitals by the mobile ECMO team and subsequently transported to the center in 2020. No disturbances in extracorporeal therapy were observed during transportation.

The parameters of the VA-ECMO therapy are presented in Table 3. The indications for VA-ECMO were as follows: myocardial infarction (4 patients), myocarditis (2 patients), intra-hospital cardiac arrest (2 patients), and high-risk percutaneous coronary intervention (3 patients). Six patients were treated with a high dose of noradrenaline before ECMO placement. Complications during VA-ECMO included left ventricle distention (4 patients), bleeding from cannulation sites (4 patients), and lower limb ischemia (2 patients).

## 4. Discussion

Our ECMO center was created over two years based solely on an existing mixed ICU in a 500 bed University Hospital in Opole. It took two and a half years for the center to operate routinely. A similar amount of time has been reported by other centers developed in tertiary hospitals [15]. The survival rates of our patients are comparable to those reported by some high-volume centers [2,12,16].

ECMO centers may be set up as a result of national healthcare strategies that involve creating network centers [17]. Having the support of local authorities is key for new ECMO center creation, particularly in its early stages [11]. Setting up our ECMO center was a totally bottom-up initiative, with financial support limited only to purchasing the ECMO machines required.

There is no consensus as to where a new ECMO center should be located [9]. Experts indicate that immediate access to various specialists should be guaranteed; thus, a tertiary hospital is considered as an optimal option [15]. In hospitals, cardiothoracic departments are often used as a location for a new ECMO center. This is above all due to the fact that extracorporeal circulation is performed routinely during open heart surgery [18]. Various types of ICUs, mainly cardiothoracic and cardiac, may be the location for a new ECMO center. A primary driving force in setting up our ECMO center was a group of intensivists who strongly felt that there would be considerable benefits. A natural consequence was, thus, to create a new ECMO center within our existing mixed ICU, and this helped us to avoid various administrative barriers related to the hospital.

Guidelines recommend that ECMO centers must meet specific criteria, including creating a team consisting of different specialists, so that various specialists are directly involved in the patient’s daily treatment. Mobile ECMO teams in these types of centers are also multidisciplinary [19,20,21].

In our center, the ECMO team is comprised only of the intensivists from the existing mixed ICU. No other specialists, such as cardiac and cardiothoracic surgeons or cardiologists, are engaged in the routine daily activity of the ECMO center. Moreover, perfusionists are not involved in the ECMO procedures at all. Therefore, we define our method of ECMO center creation as an ‘ICU-based’ approach.

In an ICU-based approach, it is assumed that an ECMO patient is usually affected by multi-organ failure, and this is comparable to other complex patients in intensive care. Consequently, and unlike in multidisciplinary-built ECMO centers, intensivists are responsible for most ECMO-related procedures, such as catheterizing vessels, priming the circuit, daily TTE/TEE examination, and routine checking of oxygenators. An ICU-based approach relieves the responsibility from external specialists, who can thus dedicate themselves to their main tasks, i.e., surgery. This model could also be used when the conditions for establishing a of multidisciplinary center are insufficient, e.g., lack of perfusionists, shortage of cardiothoracic surgeons, or intra-hospital administrative barriers. Table 4 highlights the main features of the multidisciplinary approach and our ICU-based approach to ECMO center organization.

A few studies have shown an improvement in patient outcomes after the creation of multidisciplinary ECMO team in comparison with the pre-ECMO team period. Na et al. indicated that creating a multidisciplinary team mostly contributed to favorable outcomes [22]. Hong et al. stressed the importance of their multidisciplinary approach for an improvement in outcomes of patients being treated with VA-ECMO [23]. However, several key factors in both of these studies were lacking in the pre-ECMO periods, including protocols of ECMO process, a referral system, and staff training.

Interestingly, Komindr et al. did not report any improvement in patients’ survival rate, ECMO duration, and hospital length of stay after the institution of a formal multidisciplinary ECMO team [24]. They argued that this was caused by the relatively high level of competencies in extracorporeal procedures among the staff already found during the pre-ECMO team period.

Whether the specific type of ECMO team (multidisciplinary or homogenous) matters is, thus, still not clear. The nature of the ECMO team is not necessarily the primary issue in terms of patient outcomes. Other aspects may affect the quality of care, including organized treatment, trained staff, designated leader of the team, clear protocols for ECMO inclusion, and the referral system.

Consequently, which type of ECMO center (multidisciplinary or ICU-based) is better for patients is still an unknown. Currently, a multidisciplinary approach is regarded as the ‘gold’ standard for optimum results. Notwithstanding, when extracorporeal therapy is not available at all in a region, a team consisting only of intensivists can both sufficiently perform ECMO procedures and form an efficient ECMO center within a mixed ICU. Based on our study, we also assume that intensivists can simply incorporate extracorporeal therapy into other routine modalities within the intensive care environment. A similar situation occurred in the 1990s, regarding the implementation of continuous renal replacement therapy (CRRT) in intensive care [25].

### Limitations

Our approach has several limitations. First, only involving the staff from the ICU in the center’s activity may lead to overburdening of the intensivists. Second, we do not perform extracorporeal therapy on potential donors. This limitation mainly stems from the legal situation regarding this type of therapy conducted among donors in Poland. Third, our center only performs extracorporeal therapy for the adult population. The ICU for neonates and children exists in the hospital as an entirely separate structure with dedicated staff. Fourth, for external patients only VV-ECMO is applied, by our mobile ECMO team. Except for intra-hospital eCPR, the application of VA-ECMO in the district ICU seems to be a more demanding procedure, regarding the inaccessibility of crucial facilities in district hospitals, such as a cath lab, vascular surgeon, or cardiac surgeon. Fifth, the small number of patients precludes performing both a detailed statistical analysis and an examination of the influence of various factors on the outcomes.

## 5. Conclusions

Organizing an operative ECMO center entirely on the basis of an existing mixed ICU is feasible. Although a multidisciplinary approach is still the ‘gold’ standard, an ICU-based approach can be implemented, leading to a practically functional regional ECMO center. Due to the small number of patients, further studies are needed to show that the ICU-based approach is no less effective than the multidisciplinary approach.

## Figures and Tables

**Table 1:** Baseline patient characteristics.

Variable	All Patients (*n* = 33)	VV-ECMO (*n* = 22)	VA-ECMO (*n* = 11)	*p* Value
Age, years	52 (40–59)	52 (39–58)	53 (44–68)	0.2381
Female sex, *n* (%)	8 (24.2)	4 (18.2)	4 (36.4)	0.4727
Weight, kg	80 (70–99)	85 (70–99)	72 (68–89)	0.1516
BMI, kg/cm^2^	26 (23–31)	26 (23–33)	25 (24–28)	0.1799
SOFA score	9 (7–12), *n* = 30	7 (6–12)	11 (10–14), *n* = 8	0.0051
CRRT, *n* (%)	16 (48.5)	11 (50)	5 (45.5)	0.9020
ECMO duration, hours	277 (56–464)	365 (218–496)	49 (12–113)	0.0006
ICU length of stay, days	17 (6–33)	19 (9–37)	6 (2–20)	0.0355

Note: data are presented as median (IQR), *n* (%), *n* (where data are not available for all patients). Abbreviations: BMI—body mass index, SOFA—Sequential Organ Failure Assessment, CRRT—continuous renal replacement therapy, ECMO—extracorporeal membrane oxygenation, ICU—intensive care unit.

**Table 2:** Characteristics of patients on VV-ECMO.

	All Patients (*n* = 22)	Status at 90 Days after ECMO		*p* Value
		Alive (*n* = 13)	Dead (*n* = 9)	
**Mode of circuit**				
Jugulo-femoral, *n* (%)	17 (77.3)	10 (76.9)	7 (77.8)	0.6381
Femoro-femoral, *n* (%)	5 (22.7)	3 (23.1)	2 (22.2)	0.6381
Admission cannula, Fr	25 (23–25); *n* = 15	25 (23–25); *n* = 8	25 (24–25); *n* = 7	0.6754
Return cannula, Fr	23 (22–24); *n* = 15	23 (22–25); *n* = 8	23 (21–23); *n* = 7	0.2238
**Indications for ECMO**				
ARDS of viral etiology, *n* (%)	10 (45.5)	6 (46.1)	4 (44.4)	0.4789
ARDS of bacterial etiology, *n* (%)	8 (36.4)	5 (38.4)	3 (33.3)	0.4423
ARDS of unknown etiology, *n* (%)	4 (18.2)	2 (15.4)	2 (22.2)	0.4309
**Ventilation before ECMO**				
Peak pressure, cm H_2_O	31 (29–34); *n* = 20	33 (30–35); *n* = 12	30 (28–32); *n* = 8	0.3788
Plateau pressure, cm H_2_O	29 (26–32); *n* = 16	30 (27–34); *n* = 9	26 (26–30); *n* = 7	0.3350
Driving pressure, cm H_2_O	16 (13–18); *n* = 19	16 (14–19); *n* = 11	15 (12–17); *n* = 8	0.4101
PEEP, cm H_2_O	12 (12–14)	14 (12–15)	12 (11–14)	0.1794
FiO_2_	0.8 (0.7–1)	0.8 (0.6–1)	0.8 (0.8–1)	0.3294
Tidal volume, mL/kg PBW	5 (4–5); *n* = 13	5.7 (4–6); *n* = 7	4.9 (4–5); *n* = 6	0.2644
Prone position, *n* (%)	8 (36.4)	6 (46.1)	2 (22.2)	0.4861
**Intervention on ECMO**				
Mechanical ventilation, hours	365 (179–496)	336 (168–500)	432 (215–476)	0.7528
Sedation, hours	306 (132–470); *n* = 19	306 (132–458); *n* = 11	336 (170–467); *n* = 8	0.9099
Neuromuscular blockade, *n* (%)	14 (63.6)	9 (69.2)	5 (55.5)	0.8377
Tracheostomy, *n* (%)	13 (59.1)	8 (61.5)	5 (55.5)	0.8726

Note: data are presented as median (IQR), *n* (%), *n* (where data are not available for all patients). Abbreviations: Fr—diameter in French catheter scale, ARDS—acute respiratory distress syndrome, PEEP—positive end-expiratory pressure, FiO_2_—fraction of inspired oxygen, PBW—predicted body weight, ECMO—extracorporeal membrane oxygenation.

**Table 3:** Patient characteristics on VA-ECMO.

	VA-ECMO (*n* = 11)
**Mode of circuit**	
Femoro-femoral, *n* (%)	8 (72.7)
Jugulo-femoral, *n* (%)	3 (27.3)
Admission cannula, Fr	25 (25–27); *n* = 6
Return cannula, Fr	18 (17–19); *n* = 6
**Indications for ECMO**	
Myocardial infarction, *n* (%)	4 (36.3)
Myocarditis, *n* (%)	2 (18.2)
Cardiac arrest, *n* (%)	2 (18.2)
High-risk coronary angioplasty, *n* (%)	3 (27.3)
**Parameters before ECMO**	
MAP, mm Hg	50 (35–56); *n* = 8
HR	96 (86–115); *n* = 6
EF LV, percentage	15 (5–20); *n* = 9
Creatinine, mg/dL	1.25 (0.8–1.6); *n* = 10
Arterial, pH	7.1 (7.1–7.2); *n* = 6
Lactate, mmol/L	10 (7–13); *n* = 5
**Medication before ECMO**	
Norepinephrine, μg/kg/min	0.2 (0–0.3); *n* = 10
Dobutamine, mg/kg/min	0 (0–0); *n* = 10
Adrenaline, μg/kg/min	0 (0–0); *n* = 10
**Mode of LV venting**	
IABP, *n* (%)	1 (9.1)
Atrial septostomy, *n* (%)	1 (9.1)
Impella, *n* (%)	2 (18.2)

Note: data are presented as median (IQR), *n* (%), *n* (where data are not available for all patients). Abbreviations: Fr—diameter in French catheter scale, ECMO—extracorporeal membrane oxygenation, MAP—mean arterial pressure, HR—heart rate, EF—ejection fraction, LV—left ventricle, IABP—intra-aortic balloon pump.

**Table 4:** Methods for organizing the ECMO center.

	Multidisciplinary Approach	ICU-Based Approach
Staff	Specialists from different hospital wards	Entirely ICU personnel
Leadership	Possibly a director from outside the ICU	Leader from the staff of the ICU
Who treat	Purpose-built multidisciplinary team	Intensivists, consultants only upon request
Who for	Only ECMO patients	One of the routine modalities in the ICU
Administrative structure	A separate structure in the hospital	An integral part of an existing ICU
Premises	Possibly external to the ICU	Only premises of the ICU

## Data Availability

The data presented in this study are available on request from the corresponding author.
